# Clinical and microbiological characteristics of pediatric *Pandoraea sputorum* bloodstream isolates with exploratory genomic analysis of two selected strains

**DOI:** 10.3389/fcimb.2026.1902485

**Published:** 2026-09-16

**Authors:** Lei Xi, Cen Luo, Feng Li, Yijie Zhang, Zhen Wang, Xiaomei Wang, Shiyong Deng, Yun Xiang, Changzhen Li, Xiaobin Wei

**Affiliations:** 1 Department of Laboratory Medicine, Wuhan Children’s Hospital (Wuhan Maternal and Child Healthcare Hospital), Tongji Medical College, Huazhong University of Science & Technology, Wuhan, China; 2 Department of Clinical Laboratory Medicine, Haikou Research Institute of Xiangya School of Medicine, Central South University, Haikou, Hainan, China; 3 Department of Clinical Laboratory, Hainan General Hospital (Hainan Affiliated Hospital of Hainan Medical University), Haikou, Hainan, China; 4 Wuhan Center for Disease Control and Prevention, Wuhan, China; 5 Department of Clinical Laboratory Medicine, Haikou People’s Hospital, The First Affiliated Hospital of Hainan University, Haikou, Hainan, China

**Keywords:** *bla*_OXA-1152_, comparative genomics, discordant carbapenem susceptibility, *Pandoraea sputorum*, pediatric bloodstream infection, whole-genome sequencing

## Abstract

**Background:**

*Pandoraea sputorum* (*P. sputorum*) is an uncommon opportunistic pathogen whose genomic and antimicrobial resistance features in pediatric bloodstream infection remain poorly understood. An unusual beta-lactam discordance (meropenem resistance with imipenem susceptibility) was recognized clinically but lacks a defined genomic basis.

**Methods:**

We retrospectively analyzed 34 non-duplicate *P. sputorum* isolates from pediatric blood cultures at a single center from January 2024 to February 2026. Clinical data and antimicrobial susceptibility were reviewed. Two multidrug-resistant (MDR) *P. sputorum* isolates underwent whole-genome sequencing, with species confirmed by average nucleotide identity (ANI). Comparative genomics and phylogenetic analysis used 40 P*. sputorum* genomes, including 38 public assemblies and two selected strains in our study.

**Results:**

Patients showed male predominance (70.6%) and generally mild, nonspecific presentations, mainly fever and elevated inflammatory markers. The isolates were multidrug-resistant but largely susceptible to trimethoprim-sulfamethoxazole, minocycline, levofloxacin, and ciprofloxacin. A recurrent beta-lactam discordance was observed, characterized by meropenem resistance with retained imipenem susceptibility and cefepime/ceftazidime resistance with relative ceftriaxone susceptibility. The two selected MDR isolates displaying this shared phenotype were confirmed as *P. sputorum* by whole-genome ANI analysis (>99% vs. NCTC13161). Phylogenetic analysis with 38 public *P. sputorum* genomes placed both isolates within a closely related lineage with broadly similar resistance and virulence gene profiles. Chromosomal *bla*_OXA-1152_ was identified as a leading candidate potentially associated with carbapenem discordance, whereas additional beta-lactamase-related genes, efflux systems, porin/outer-membrane candidates, and PBP-associated targets were identified and support a multifactorial resistance background. The *adeF*-associated RND efflux locus may also contribute to multidrug resistance, but functional validation is required.

**Conclusions:**

This study describes the clinical and microbiological characteristics of pediatric *P. sputorum*-positive blood cultures at a single center and provides exploratory genomic data for two selected isolates. The recurrent carbapenem-discordant phenotype warrants careful antimicrobial susceptibility interpretation, and comprehensive molecular typing and functional studies are needed to define its genomic basis.

## Introduction

The genus *Pandoraea* was first proposed by Coenye et al. in 2000 (Coenye et al., 2000) and comprises aerobic, non-spore-forming, non-glucose-fermenting Gram-negative bacilli that are phylogenetically related to *Burkholderia* and *Ralstonia*-related taxa (Pimentel and MacLeod, 2008; Munson and Carroll, 2023; Carella et al., 2025). Members of this genus are widely distributed in environmental niches such as soil, water, and the rhizosphere, where they participate in organic compound degradation and bioremediation (Jin et al., 2007; Höger et al., 2016; Peeters et al., 2016). Although initially regarded mainly as environmental bacteria, *Pandoraea* species have increasingly been recognized in clinical microbiology as rare opportunistic pathogens (Saiman et al., 2024; Herzog et al., 2025).

Clinically, *Pandoraea* species have been most frequently reported in patients with cystic fibrosis (CF) (Davies and Rubin, 2007; Bayjanov et al., 2019; Reshad et al., 2024), particularly in association with chronic airway colonization and respiratory infection (Atkinson et al., 2006; Coward et al., 2020; Pither et al., 2021). Previous studies have also suggested that *Pandoraea* spp. may invade epithelial cells and induce pro-inflammatory cytokines, supporting their potential pathogenicity rather than simple colonization in selected clinical contexts (Caraher et al., 2008). Beyond CF, *Pandoraea* species have also been recovered from patients with chronic obstructive pulmonary disease (Herzog et al., 2025), trauma (Lin et al., 2019; Ma et al., 2022), immunosuppressive conditions or populations (Xiao et al., 2019), and bloodstream infections (Itoh et al., 2022; Ramos Oliveira et al., 2023), and patient-to-patient transmission has been suggested in medical care settings (Degand et al., 2015; Kruis et al., 2023). Several species, including *P. apista* (Atkinson et al., 2006; Lin et al., 2019; Itoh et al., 2022), *P. pnomenusa* (Stryjewski et al., 2003; Ambrose et al., 2016; Ramos Oliveira et al., 2023), *P. pulmonicola* (Dupont et al., 2017; Pither et al., 2021), and *P. sputorum* (Xiao et al., 2019; Reshad et al., 2024), have been isolated from both CF and non-CF specimens. Although the number of documented infections remains relatively limited, severe outcomes such as sepsis, multiple organ failure, and death have been reported, particularly in invasive infections caused by *P. pnomenusa* and other clinically relevant *Pandoraea* species (Stryjewski et al., 2003; Stryjewski et al., 2003; Bodendoerfer et al., 2021; Ma et al., 2022; Halabi et al., 2025). Recently, genomic epidemiological work from China further highlighted *Pandoraea* spp. as emerging bloodstream pathogens in non-CF populations and emphasized the value of whole-genome sequencing (WGS) for species identification and surveillance (Chen et al., 2025). However, systematic data on pediatric-derived isolates of *P. sputorum* remain limited.

Accurate identification of *Pandoraea* spp. is challenging because conventional phenotypic systems may misidentify uncommon non-fermenting Gram-negative bacilli as *Burkholderia*, *Ralstonia*, or *Stenotrophomonas* species (McMenamin et al., 2000; Bosshard et al., 2006; Pimentel and MacLeod, 2008). Although 16S rRNA and *gyrB* sequencing can assist species identification, their resolution may be insufficient for closely related *Pandoraea* taxa, whereas WGS and average nucleotide identity (ANI)-based comparative genomic analyses have improved the understanding of *Pandoraea* taxonomy, phylogeny, and functional potential (Bayjanov et al., 2019; Peeters et al., 2019; Gao et al., 2022). Previous studies of CF-associated *Pandoraea* strains have shown that several isolates assigned to recognized species, including *P. sputorum*, may represent novel taxa, underscoring the importance of WGS-based species identification (Mia et al., 2026). Comparative genomic analyses have also identified environmental or plant-associated isolates as *P. sputorum* using ANI, AAI, MLSA, and genome-to-genome distance approaches, and have reported selected functional traits such as quorum sensing systems and OXA-type beta-lactamases (Gao et al., 2022). More recent genomic analyses of bloodstream infection-associated *P. sputorum* isolates suggested the emergence of clonal lineages carrying core genome-encoded resistance determinants, including efflux pump genes and OXA-type beta-lactamases (Wu et al., 2026). However, systematic clinical genomic data for pediatric bloodstream *P. sputorum* isolates remain limited, and the relationship between genome-encoded resistance candidates has not been well explored.

Another challenge is the unusual antimicrobial susceptibility profile of *Pandoraea* spp. Intrinsic carbapenem-hydrolyzing oxacillinases have been described in this genus, and variable susceptibility to beta-lactams, carbapenems, aminoglycosides, and other antimicrobial classes has been reported (Schneider and Bauernfeind, 2015). Previous studies have suggested that *P. sputorum* may exhibit intrinsic or genome-encoded resistance to multiple antimicrobial classes. Notably, Zhang et al. recently reported an unusual beta-lactam susceptibility pattern in *P. sputorum*, characterized by meropenem resistance with retained imipenem susceptibility, and linked this discordance to the oxacillinase variant OXA-1152 (Zhang et al., 2025). This intra-class discordance is clinically relevant and raises questions regarding its underlying genomic basis.

To address these gaps, we conducted a retrospective clinical and microbiological study of 34 non-duplicate *P. sputorum* isolates recovered from pediatric blood cultures. Their clinical features and antimicrobial susceptibility profiles were analyzed, and two MDR isolates were selected for WGS. By incorporating 38 publicly available *P. sputorum* genome assemblies, we constructed a SNP-based phylogenetic framework encompassing 40 genomes and examined the distribution of resistance-associated and virulence-associated genes. This integrated approach aimed to characterize the clinical significance, population structure, and candidate genomic determinants of antimicrobial resistance in *P. sputorum*, with particular emphasis on the meropenem-resistant/imipenem-susceptible phenotype.

## Materials and methods

### Strain collection and identification

A total of 34 non-duplicated *P. sputorum* strains were recovered from positive blood cultures of 34 pediatric patients at our center between January 2024 and February 2026. Only the first isolate from each patient was included. Blood cultures were collected as part of routine clinical care when bloodstream infection was clinically suspected. For each culture, 3–5 mL of peripheral venous blood was aseptically obtained by venipuncture and inoculated into blood culture bottles. The bottles were then incubated using automated blood culture system (BACTEC FX, BD, USA). A positive blood culture was defined as a blood culture bottle flagged as positive by the automated blood culture system, followed by recovery of bacterial growth on subculture and subsequent identification as *P. sputorum*.

When a blood culture bottle flagged positive, the positive broth was subcultured onto blood agar, MacConkey agar, and chocolate agar plates (Zhongjin Technology Co., Ltd., Wuhan, China) and incubated at 37 °C with 5% CO_2_ for 18–24 h. Pure colonies were identified using an IVD MALDI Biotyper system equipped with a Microflex LT/SH instrument (Bruker Daltonik GmbH, Bremen, Germany). Spectra were acquired using flexControl software version 3.4 (Build 85.1) within Compass for flexSeries 1.4 and analyzed using MALDI Biotyper software version 3.1 (Build 65). The Bruker Foundation Library version 4.7.373.0 was used for spectral comparison. According to the manufacturer’s criteria, a score of ≥ 2.0 was considered reliable for species-level identification, whereas scores between 1.70 and 1.99 were considered reliable for genus-level identification. Given the limited representation of *Pandoraea* species in the MALDI-TOF MS database and the potential for misidentification, species identity of the two sequenced isolates was further confirmed by WGS and ANI analysis (Bosshard et al., 2006). All isolates were stored at −80 °C in skim milk for further use.

### Clinical data collection and primary epidemiological investigation

Clinical data were retrospectively collected from the electronic medical record system. The 34 isolates were included for clinical, microbiological, antimicrobial susceptibility, and descriptive epidemiological analyses, whereas WGS-based conclusions were restricted to the two sequenced isolates. Specifically, the collected information included demographic characteristics, underlying diseases, admission and discharge dates, blood culture collection dates, microbiological report dates, ward/campus location, laboratory findings, treatment, and outcomes. For descriptive epidemiological analysis, the timing of isolate recovery was assessed according to the interval between hospital admission and blood culture collection. Isolates recovered from blood cultures collected on the day of admission or within 48 h after admission were classified as admission-onset detections, whereas isolates recovered from blood cultures collected more than 48 h after admission were classified as hospital-onset detections. This study was approved by the Medical Ethics Committee of Wuhan Children’s Hospital (No. 2024R050-E03). Informed consent was waived due to the retrospective nature of the study and the use of anonymized data.

### Antimicrobial susceptibility testing

Minimum inhibitory concentrations (MICs) were determined using the broth microdilution method with commercial 96-well microtiter plates (GN4F plate, Sensititre; Thermo Fisher Scientific, USA). Bacterial suspensions (0.5 McFarland) were prepared in cation-adjusted Mueller-Hinton broth with TES (N-tris(hydroxymethyl)methyl-2-aminoethanesulfonic acid) and inoculated into the plates. After incubation at 37 °C for 18–24 hours, results were read visually. Because species-specific clinical breakpoints for *P. sputorum* are not well established for all tested agents, the antibiotic susceptibility pattern was characterized according to the standards established for other non-*Enterobacterales* by the Clinical and Laboratory Standards Institute (CLSI M100, 36th Edition) (Zhang et al., 2025; Clinical and Laboratory Standards Institute, 2026). Results were interpreted cautiously and reported as susceptible, intermediate, or resistant according to these surrogate criteria. Cefoperazone breakpoints were applied for interpreting cefoperazone-sulbactam (SCF) results (Jones et al., 1987). *Escherichia coli* ATCC 25922 and *Pseudomonas aeruginosa* ATCC 27853 were used as quality control strains. MDR was defined as acquired non-susceptibility to at least one agent in three or more antimicrobial categories. In this study, carbapenem susceptibility discordance mainly referred to inconsistent susceptibility results between meropenem and imipenem.

### DNA extraction and WGS

Due to resource constraints and the incomplete availability of all archived isolates for further subculture, two selected isolates, *P. sputorum* whet-A341 and *P. sputorum* whet-A408 (hereafter abbreviated as *P. sputorum*-341 and *P. sputorum*-408), were subjected to WGS. These two isolates were selected because they (i) exhibited a MDR phenotype observed among the isolates, (ii) were isolated at different time points, and (iii) were recovered from patients of different age groups. WGS-based conclusions were restricted to the two sequenced isolates and were not extrapolated to the full collection of 34 isolates.

Genomic DNA of *P. sputorum*-341 and *P. sputorum*-408 was extracted from overnight bacterial cultures using the Bacterial Genomic DNA Extraction Kit (TIANLONG Tech, Xi’an, China) according to the manufacturer’s instructions. Both short-read and long-read sequencing were performed for the two isolates. Short-read sequencing was conducted using a paired-end 150-bp strategy, and raw short-read data were quality-filtered using fastp v0.23.4. Long-read sequencing was performed on the PromethION platform (Oxford Nanopore Technologies, Oxford, UK) using R10.4.1 flow cells. The detailed sequencing and quality control data of *P. sputorum*-341 and *P. sputorum*-408 were provided in **Supplementary Table 1**. Taxonomic classification of both short-read and long-read data was performed using Kraken2 to assess potential contamination. For *P. sputorum*-341, *P. sputorum* accounted for 95.64% of classified short reads and 96.63% of classified long reads. For *P. sputorum*-408, *P. sputorum* accounted for 95.45% of classified short reads and 96.42% of classified long reads. These results supported that *P. sputorum* was the predominant organism in the sequencing data of both isolates.

### Bioinformatics and comparative genomics

Hybrid *de novo* genome assembly was performed using Unicycler v0.4.8 (Wick et al., 2017) with both Illumina short reads and ONT long reads. Short reads were used to improve base-level accuracy, whereas long reads were used to improve assembly contiguity and resolve repetitive regions. Assembly graphs and circularization status were inspected using Bandage. Assembly quality was evaluated based on genome size, GC content, number of contigs, N50, largest contig, sequencing depth, and circularization status. Assembly statistics of the two sequenced isolates are summarized in **Supplementary Table 1**. Average nucleotide identity (ANI) was calculated between our isolates and reference *Pandoraea* genomes from the NCBI database using JSpeciesWS, with a threshold of ≥ 95% ANI for species-level delineation (Goris et al., 2007; Richter and Rosselló-Móra, 2009).

Gene prediction and functional annotation were performed using Prokka v1.14 (Seemann, 2014). Predicted coding sequences (CDSs) were further annotated against multiple databases, including NCBI NR, Swiss-Prot, COG, KEGG, and GO. Antimicrobial resistance-associated genes were identified using the Comprehensive Antibiotic Resistance Database (CARD v3.2.7) (Alcock et al., 2020) with RGI, and hits classified as “perfect” or “strict” were retained for interpretation, whereas “loose” hits were manually reviewed and interpreted cautiously. Virulence-associated factors were identified using the Virulence Factor Database (VFDB) (Liu et al., 2019) with thresholds of ≥ 80% sequence identity and ≥ 70% query coverage, followed by manual curation. Insertion sequences and transposable elements were screened using the ISfinder online database (Siguier et al., 2006). Prophage regions were predicted using PhiSpy, and CRISPR arrays were identified using CRISPRCasFinder. Genomic islands were predicted using Alien Hunter. The overall genome architecture was visualized using CGView. The WGS findings apply only to the two sequenced isolates.

A total of 43 publicly available *P. sputorum* genome assemblies were initially retrieved from the European Nucleotide Archive (ENA). After excluding duplicate entries and low-quality assemblies (N50 < 50 kb or completeness < 90%), 38 high-quality assemblies were retained and combined with our two newly sequenced isolates, yielding a final dataset of 40 genomes for comparative phylogenetic analysis. For comparative genomic analysis, all genomes in the final dataset were reanalyzed using a unified bioinformatics workflow. To ensure comparability, all 40 genomes were subjected to the same pipeline for gene prediction, functional annotation, resistance gene screening, virulence factor detection, orthologous gene clusters (OrthoVenn3), and pangenome analysis (Roary v3.13.0) (Page et al., 2015) with a BLASTP identity threshold of 95%. Furthermore, a core-genome alignment-based phylogenetic tree was constructed from the Roary core-gene alignment using RAxML (Stamatakis, 2014) and visualized together with resistance gene and virulence factor heatmaps using iTOL (Interactive Tree of Life, v6). Multiple sequence alignments and secondary structural annotations of key resistance-associated proteins were performed using ESPript 3.0.

## Results

### Mild and nonspecific pediatric bloodstream presentations defined the clinical context

During the study period, 34 non-duplicate *P. sputorum* strains were isolated from blood cultures of 34 individual pediatric patients. The cohort exhibited a male predominance, accounting for 70.6% (24/34). The patients spanned a wide age range, from neonates to adolescents up to 17 years old. The clinical features of representative cases are presented in **Table 1**. For instance, a 9-year-old boy infected by strain *P. sputorum*-408 presented with a 4-day history of cough and a 1-day history of vomiting. Laboratory tests showed an elevated white blood cell count (11.93 × 10^9^/L) with a high neutrophil percentage (91.4%) and increased C-reactive protein (36.7 mg/L). His blood culture was positive for *P. sputorum* at 26 hours. Following treatment with cefmetazole and azithromycin, his symptoms resolved. Overall, the clinical manifestations in our cohort were relatively mild, mostly presenting as fever with elevated inflammatory markers, without severe sepsis or septic shock.

**Table 1:** Clinical characteristics and laboratory infection-related indicators of six pediatric patients with bloodstream *P. sputorum* strains.

Strain No.	Age	Sex	TTP (h)	Main complaint	T (°C)	Fever Duration (d)	ESR (mm/h)	PCT (ng/mL)	hsCRP (mg/L)	WBC (×10^9^/L)	Neu (×10^9^/L)	Neu%
*P. sputorum*-424	11y11m	M	24.7	Fever and cough for 4d	40.4	9	/	0.55	46.1	6.75	5.08	75.2
*P. sputorum*-426	1d	M	25.0	Respiratory distress for 13h after birth	37.4	5	/	/	0.3	14.59	10.58	72.5
*P. sputorum*-423	6y7m	M	22.5	Rhabdomyosarcoma for 4y, fever for 2d	38.4	11	/	0.58	113	0.24	0.02	8.4
*P. sputorum*-408	9y10m	M	26.0	Cough for 4d, vomiting for 1d	38.1	1	13	0.24	36.7	11.93	10.9	91.4
*P. sputorum*-852	9y	M	22.5	Fever for 2d	40	3	42	0.15	83	12.18	8.8	72.2
*P. sputorum*-341	8y6m	F	20.0	Cough for 7d, persistent fever for 3d	38	7	/	0.06	8.83	8.36	5.28	63.1

y, year; m, month; d, day; h, hour; M, male; F, female; TTP, time to positivity; T (°C), highest body temperature on the day of blood culture positivity; ESR, erythrocyte sedimentation rate; PCT, procalcitonin; hsCRP, hypersensitive C-reactive protein; WBC, white blood cell count; Neu, neutrophil count; Neu%, neutrophil percentage.

To further evaluate the dynamics of bloodstream infection, we analyzed the blood culture time to positivity (TTP), which serves as a surrogate marker for initial bacterial load. A weak but statistically significant positive correlation was observed between patient age and TTP (Spearman R = 0.34, P = 0.048) (**Figure 1A**), suggesting that younger patients tended to have a slightly shorter TTP. However, when stratified into four clinical age groups (neonate, infant, child, and older child), no statistically significant difference in TTP was detected (Kruskal-Wallis, P = 0.57) (**Figure 1B**). This likely reflects the limited sample size within each subgroup, which restricted the statistical power to detect potential age-related differences. Nevertheless, the overall correlation analysis and the marginal significance of the Spearman test suggest that age-related variation in bacteremia burden may warrant further investigation in larger cohorts.

**Figure 1:**
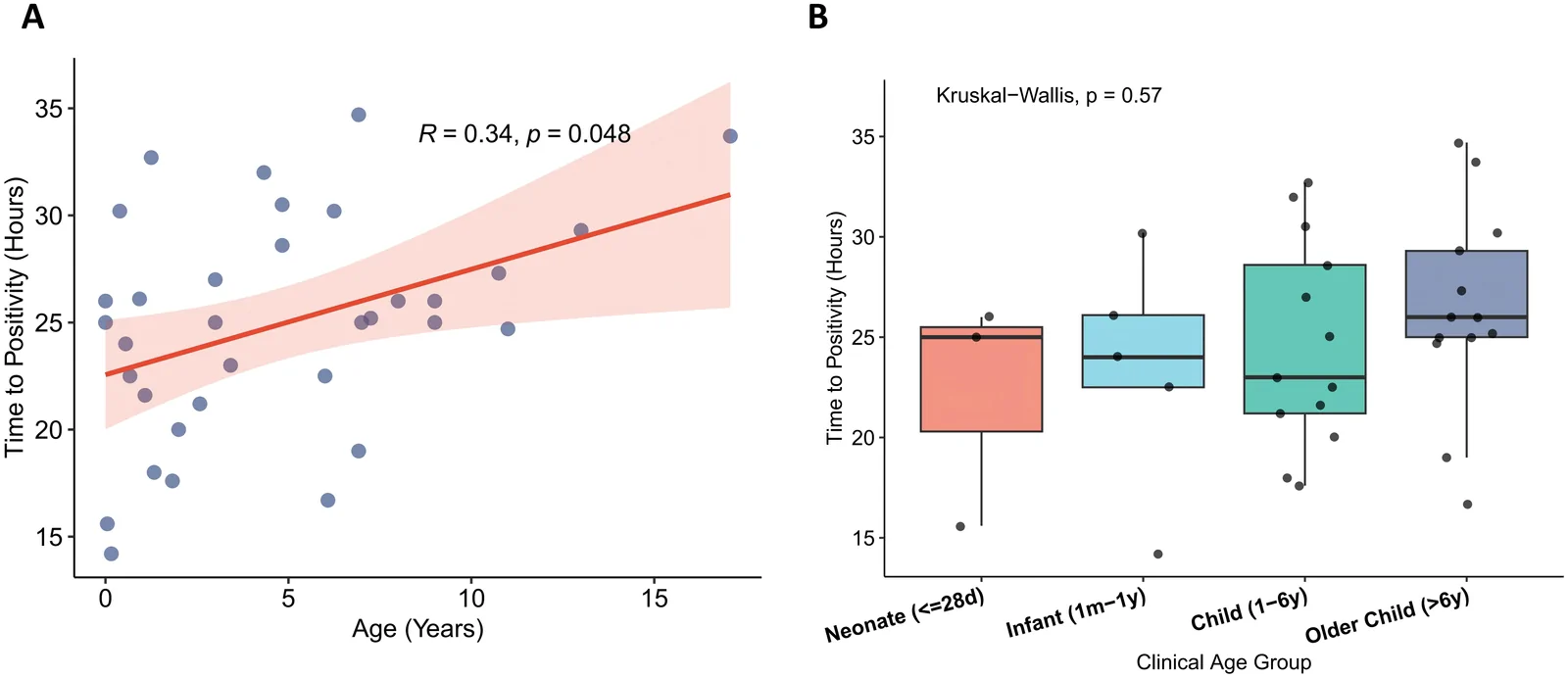
Clinical dynamics of *P. sputorum* bloodstream isolates. **(A)** Correlation analysis between patient age and TTP (Spearman R = 0.34, P = 0.048). **(B)** Comparison of TTP across different clinical age groups (Kruskal-Wallis, P = 0.57).

Given the uncommon recovery of *P. sputorum* from pediatric blood cultures, we further reviewed the available epidemiological information to assess whether the cases showed features suggestive of potential healthcare-associated transmission. Admission dates, discharge dates, blood culture collection dates, report dates, and hospital wards were reviewed for all 34 patients. The isolates were recovered over approximately 2 years from January 2024 to February 2026, rather than within a short outbreak window. These cases were widely distributed across 12 different wards. Pediatric Gastroenterology Ward II accounted for the largest number of cases (8/34), but these cases occurred intermittently over a prolonged period and did not form a short-term ward-specific cluster. We further assessed the timing of blood culture collection relative to hospital admission. Most blood cultures were collected on the day of admission or within 48 h after admission (32/34, 94.1%), whereas only two cases (2/34, 5.9%) were classified as hospital-onset detection based on blood culture collection more than 48 h after admission (**Supplementary Table 2**). Overall, based on the available temporal, ward-distribution, and hospitalization-timing data, we did not identify clear evidence of a short-term single outbreak or a typical point-source healthcare-associated transmission event.

### Pediatric bloodstream isolates shared a recurrent MDR and carbapenem-discordant phenotype

The antimicrobial susceptibility profiles of the 34 isolates revealed a high MDR rate. As shown in **Figure 2A**, most strains were resistant to multiple classes of antibiotics. However, the strains generally remained susceptible to trimethoprim-sulfamethoxazole (SXT), minocycline (MIN), levofloxacin (LVX), and ciprofloxacin (CIP) (**Figure 2B**). A distinctive intra-class discordance was observed among beta-lactam antibiotics. The isolates were highly resistant to meropenem (MEM) but remained susceptible to imipenem (IPM). A similar divergence was observed within cephalosporins, where the isolates were predominantly resistant to cefepime (FEP) and ceftazidime (CAZ), yet showed susceptibility or intermediate resistance to ceftriaxone (CRO) (**Figure 2C**). Heatmap analysis revealed that this susceptibility signature was consistently conserved across isolates collected throughout the study period, suggesting a stable and recurrent susceptibility pattern. This pattern is compatible with intrinsic or chromosome-encoded mechanisms, although this possibility could not be confirmed without genomic analysis of all isolates.

**Figure 2:**
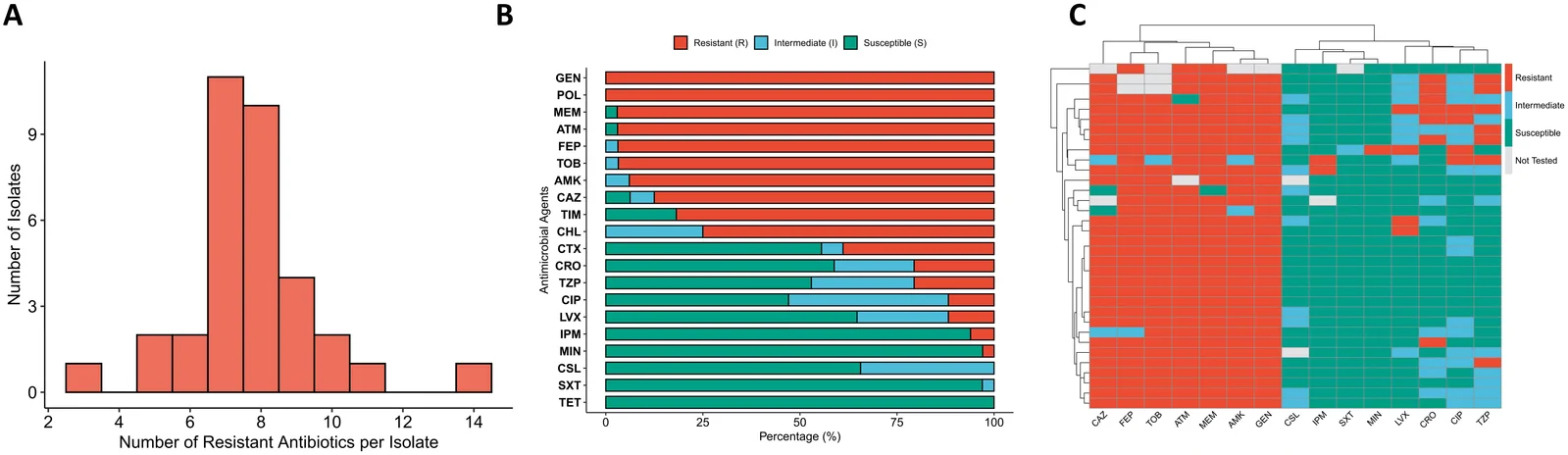
Antimicrobial susceptibility profiles of the 34 clinical *P. sputorum* isolates. **(A)** Distribution of MDR burden among the isolates. **(B)** Overall susceptibility, intermediate, and resistance rates to tested antimicrobial agents. **(C)** Heatmap illustrating the intra-class discordance in susceptibility to beta-lactam antibiotics.

### Two selected MDR isolates with the shared phenotype were confirmed as *P. sputorum*

The clinical and microbiological analyses identified a conserved susceptibility pattern across the pediatric bloodstream isolate collection. All 34 isolates showed meropenem resistance with retained imipenem susceptibility, together with resistance to cefepime and ceftazidime but relative susceptibility to ceftriaxone. Because this recurrent phenotype was clinically relevant and compatible with a stable resistance background, two MDR isolates, *P. sputorum*-341 and *P. sputorum*-408, were selected for WGS. These two isolates displayed the same carbapenem-discordant phenotype observed in the overall collection and were therefore used as selected strains to explore candidate genome-encoded determinants. Notably, the genomic analyses described below are therefore based on these two isolates only. The complete genome of *P. sputorum*-341 was assembled into a single chromosome of 5,902,068 bp (~5.90 Mb) with a GC content of 62.71%, containing 5,223 CDSs, whereas *P. sputorum*-408 possessed a genome of 5,940,500 bp (~5.94 Mb) with a GC content of 62.69% and 5,279 CDSs. The overall genome architecture of *P. sputorum*-341, including CDS distribution, GC content, GC skew, rRNA/tRNA loci, and genomic islands, is presented in **Figure 3A**. Species identity was confirmed at the whole-genome level. The ANI values between the two clinical isolates and the reference *P. sputorum* strain NCTC13161 were 99.05% and 99.00%, respectively, both well above the accepted species delineation threshold of 95%. Consistently, annotation against the NR database showed that the majority of sequences were assigned to *P. sputorum*, accounting for 80.41% of all annotated hits (**Figure 3B**). Together, these results confirmed that both sequenced clinical isolates belong to *P. sputorum*.

**Figure 3:**
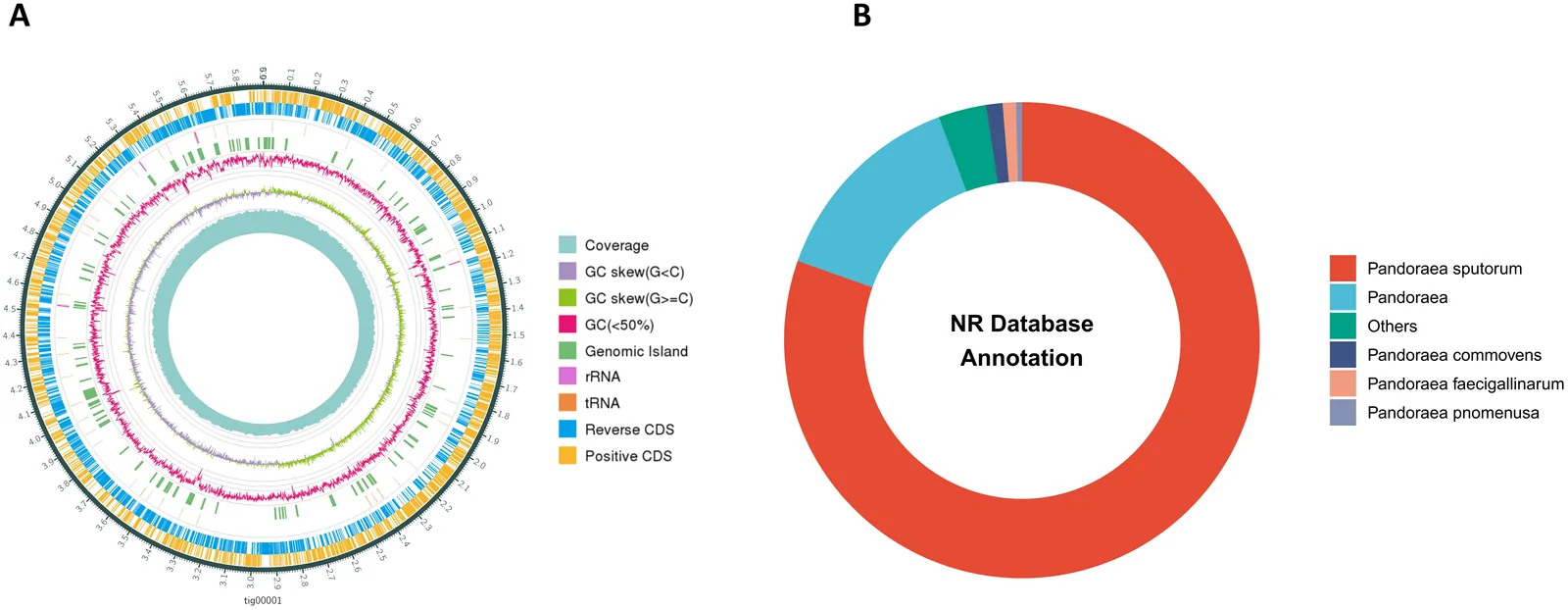
Genome features and species assignment of the clinical *P. sputorum* isolates. **(A)** Circular genome map of *P. sputorum*-341, showing from outer to inner rings: positive-strand CDSs, negative-strand CDSs, tRNA genes, rRNA genes, genomic islands, GC content, GC skew, and sequencing coverage. **(B)** Taxonomic distribution of NR database annotations for the predicted genes.

### Comparative genomics placed the two isolates within a conserved *P. sputorum* genomic background

Functional annotation of predicted CDSs of the two sequenced isolates against the COG, GO, and KEGG databases revealed a genomic repertoire typical of environmental beta-proteobacteria, with strong representation of categories related to amino acid transport and metabolism, transcription, inorganic ion transport, membrane transport, and signal transduction (**Figure 4**). Notably, genes associated with defense mechanisms (110 CDSs by COG) and membrane transport (235 CDSs by KEGG) were well represented, consistent with the MDR phenotype observed phenotypically. To explore intraspecies diversity, comparative genomic analysis was performed using the two sequenced clinical isolates together with four representative *P. sputorum* strains selected based on the SNP-based phylogenetic framework, including two closely related strains (B10 and B01) and two more distantly related strains (WP46 and EFB03792). The UpSet plot revealed a conserved core genome composed of 3,898 orthologous gene clusters shared by all six strains (**Figure 5**), highlighting a highly stable genomic backbone within the species. In addition, 337 gene clusters were shared exclusively among the closely related clade consisting of strains 341, 408, B10, and B01, indicating lineage-associated genomic features. Strain-specific gene content was also observed: *P. sputorum*-341 and *P. sputorum*-408 carried 27 and 26 unique gene clusters, respectively. These accessory and unique gene sets may reflect ongoing genome diversification and could contribute to strain-specific ecological adaptation or clinical behavior.

**Figure 4:**
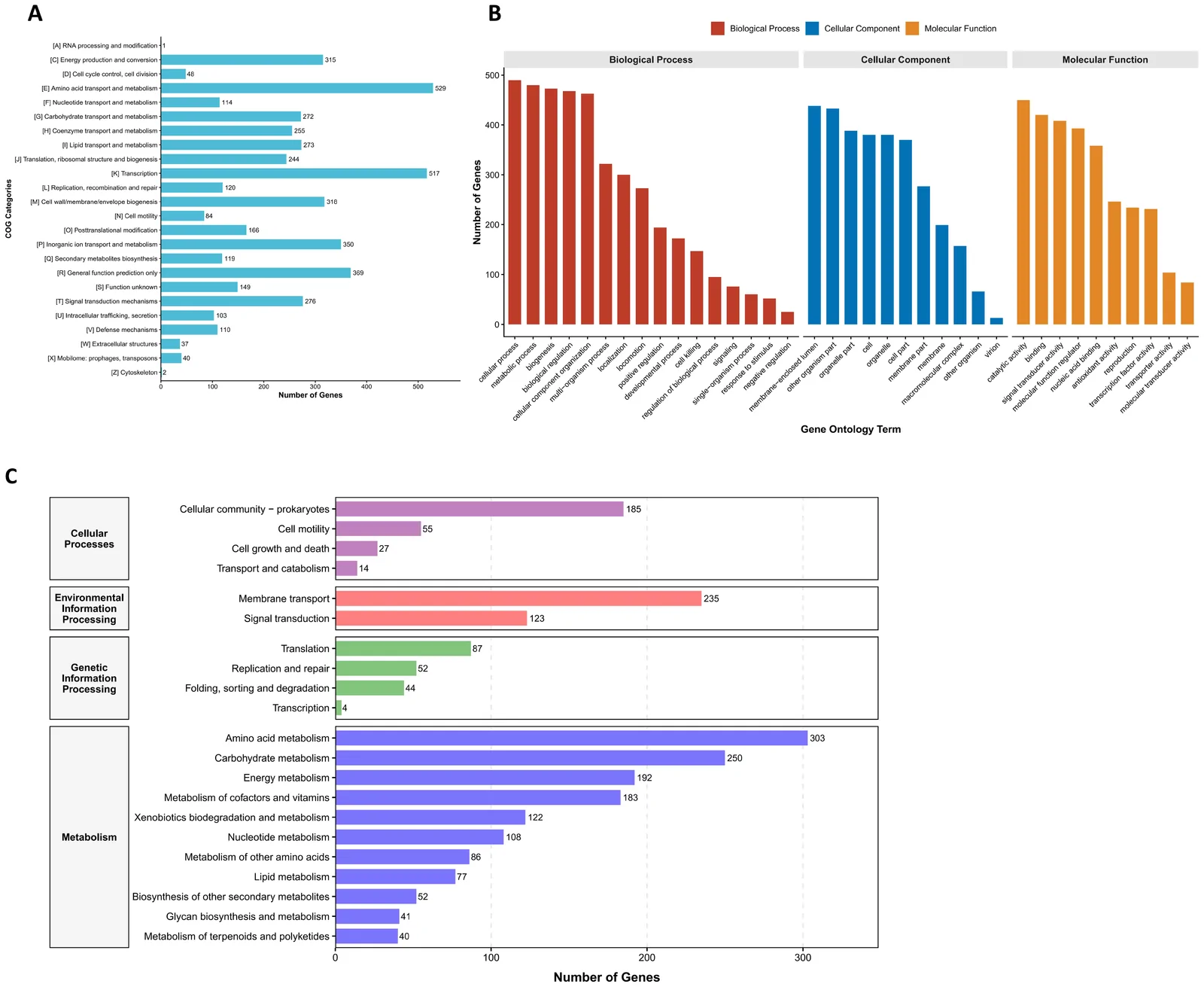
Functional annotation of the *P. sputorum* clinical isolate genome based on COG **(A)**, GO **(B)**, and KEGG databases **(C)**.

**Figure 5:**
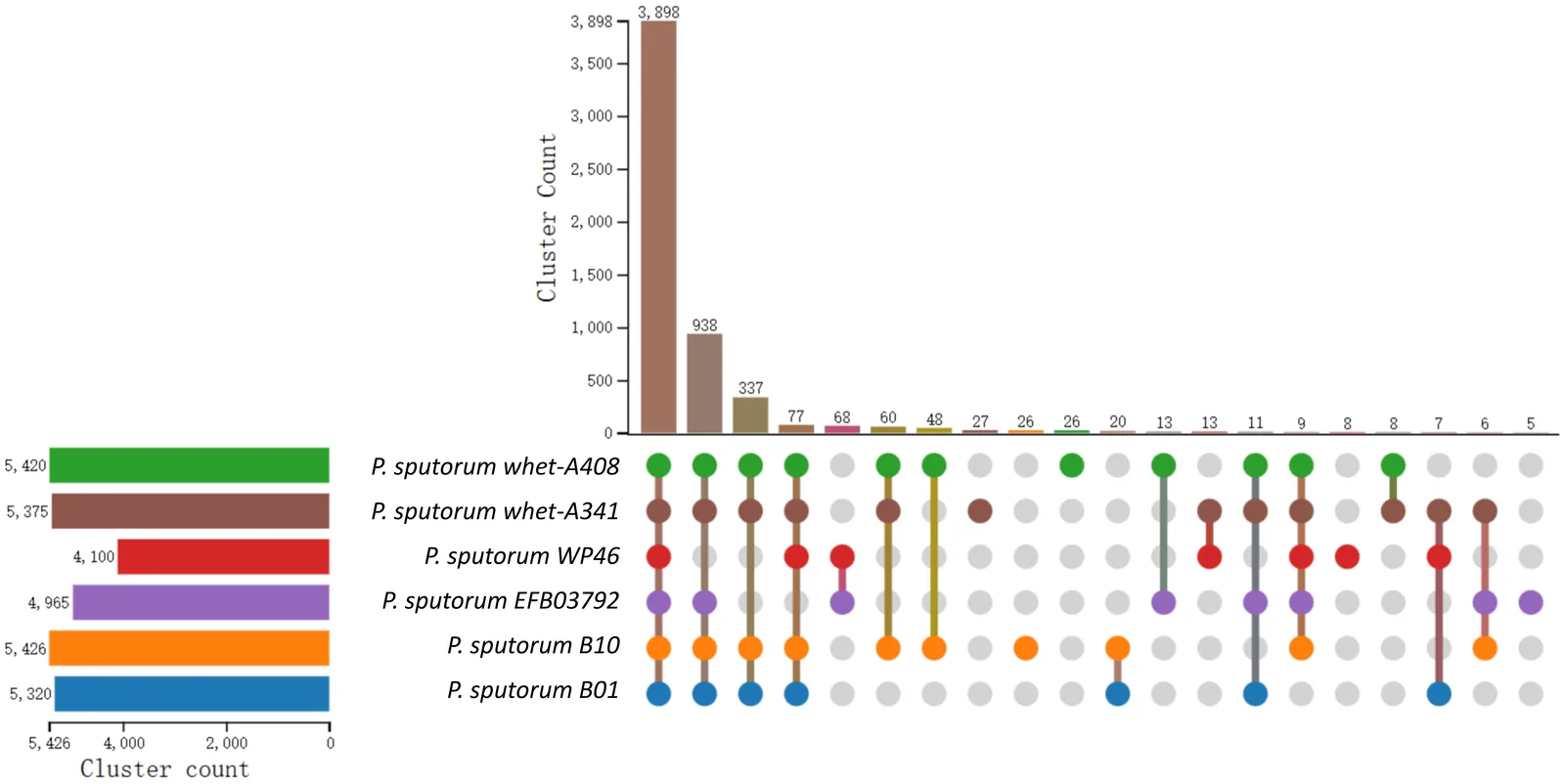
Comparative pangenome analysis of six *P. sputorum* strains. UpSet plot showing the distribution of shared and unique orthologous gene clusters among six *P. sputorum* strains.

### Phylogenetic analysis linked the two isolates to *bla*_OXA-1152_-positive strains

To place these two isolates into a broader evolutionary framework, we constructed a SNP-based phylogenetic tree using 40 P*. sputorum* genome sequences and integrated the distribution patterns of major virulence-associated and antimicrobial resistance genes (**Figure 6**). The phylogenetic analysis showed that *P. sputorum*-341 and *P. sputorum*-408 clustered tightly together within a closely related lineage. Both strains were also closely related to previously reported *P. sputorum* strains carrying *bla*_OXA-1152_ (from *P. sputorum*-B01 to *P. sputorum*-B12) (Zhang et al., 2025). This is noteworthy because a recent functional study demonstrated that *bla*_OXA-1152_ contributed to meropenem resistance in closely related *P. sputorum* strains: deletion of *bla*_OXA-1152_ reduced the meropenem MIC and restored susceptibility, and *bla*_OXA-1152_ was confirmed to hydrolyze meropenem more efficiently than imipenem (Zhang et al., 2025). Therefore, the close phylogenetic relationship between our isolates and the *bla*_OXA-1152_-positive strains suggests that a similar resistance background may exist in this lineage. The resistance gene heatmap further showed that most isolates in this lineage shared a highly conserved resistance gene repertoire, including genes related to beta-lactam resistance and efflux function (**Figure 6**, right panel). Virulence gene profiles were also largely conserved, with limited variation among sublineages (**Figure 6**, middle panel). These findings indicate that *P. sputorum*-341 and *P. sputorum*-408 belong to a closely related branch with a broadly similar resistance-associated gene content.

**Figure 6:**
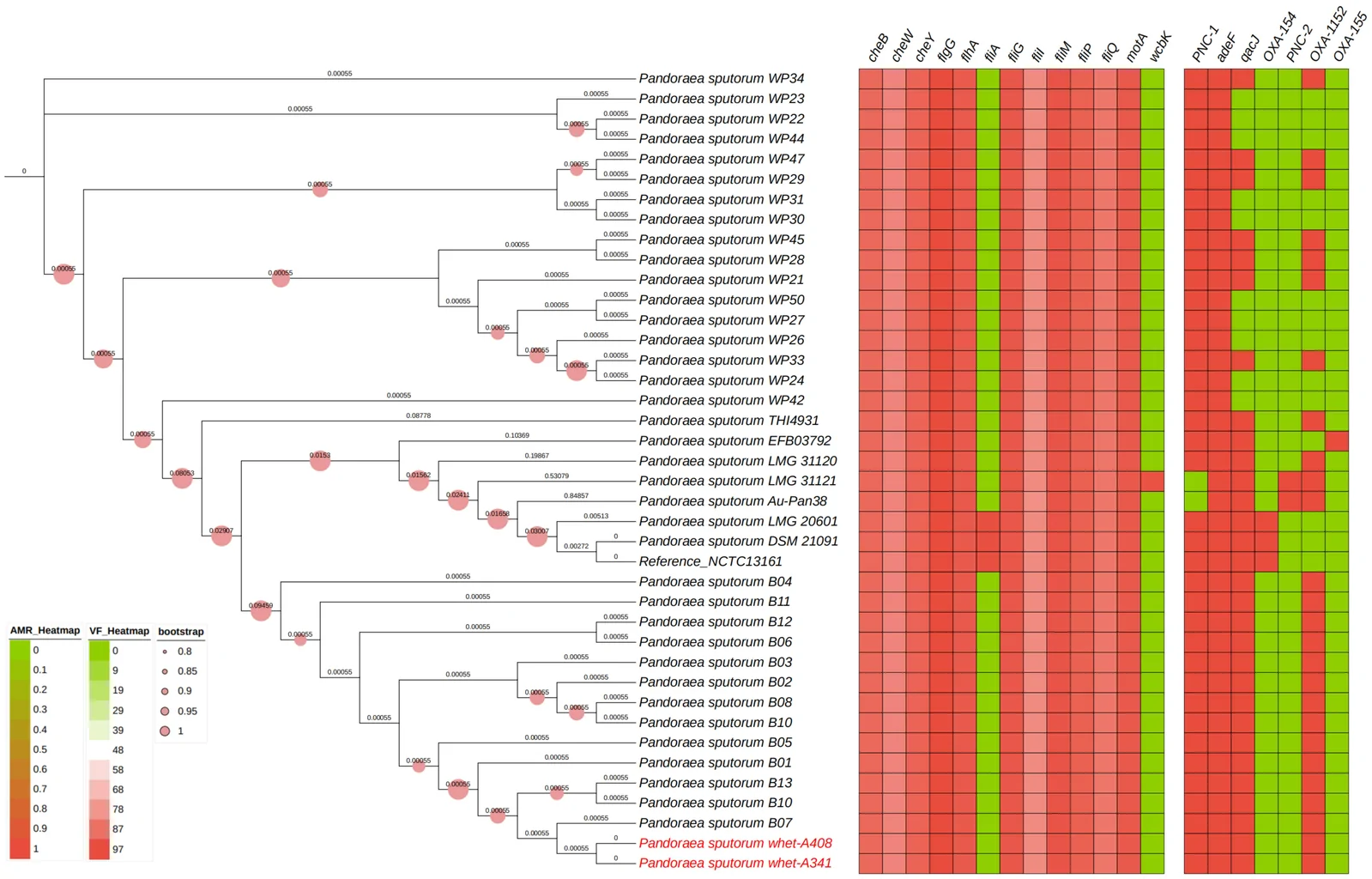
Comprehensive phylogenetic and multi-omics profiling of 40 P*. sputorum* genomes. The phylogenetic tree is combined with heatmaps showing the presence or absence of key antimicrobial resistance genes and virulence factors.

### Genome annotation identified *bla*_OXA-1152_ and additional candidates supporting a multifactorial resistance background

To investigate the genetic basis of the meropenem-resistant phenotype of *P. sputorum*-341 and *P. sputorum*-408, genome-encoded candidate features related to resistance phenotypes were annotated and summarized in **Supplementary Table 3**. Both sequenced isolates showed highly conserved candidate profiles, including beta-lactamase-related candidates, efflux-associated systems, porin or outer membrane-related proteins, and penicillin-binding proteins (PBPs).

The OXA-type beta-lactamase hit was further supported by BLAST analysis as *bla*_OXA-1152_. The *bla*_OXA-1152_-containing chromosomal region was then extracted and its local genetic environment was analyzed. Structural comparison did not identify any typical insertion sequence, transposase, or other recognizable mobile genetic elements flanking this gene (**Figure 7A**). All matched IS elements from the ISfinder database exhibited non-significant E-values above the conventional cutoff of 1e-5 (**Figure 7B**), indicating that no reliable IS element was detected in this region. These results suggest that *bla*_OXA-1152_ in our isolates is maintained as a chromosomal component rather than being embedded within a recognizable mobile genetic cassette. Together with the functional evidence reported for closely related *P. sputorum* strains (Zhang et al., 2025), these data support the possibility that *bla*_OXA-1152_ may represent a leading candidate potentially associated with the meropenem resistance phenotype in *P. sputorum*-341 and *P. sputorum*-408, although direct functional validation in these specific isolates was not performed. OXA-type class D beta-lactamases may exhibit substrate-dependent hydrolytic profiles, and different catalytic efficiencies toward meropenem and imipenem could theoretically contribute to the observed discordant phenotype. However, enzyme kinetic assays using purified OXA-1152 were not performed in this study; therefore, substrate-specific hydrolytic activity remains unconfirmed.

**Figure 7:**
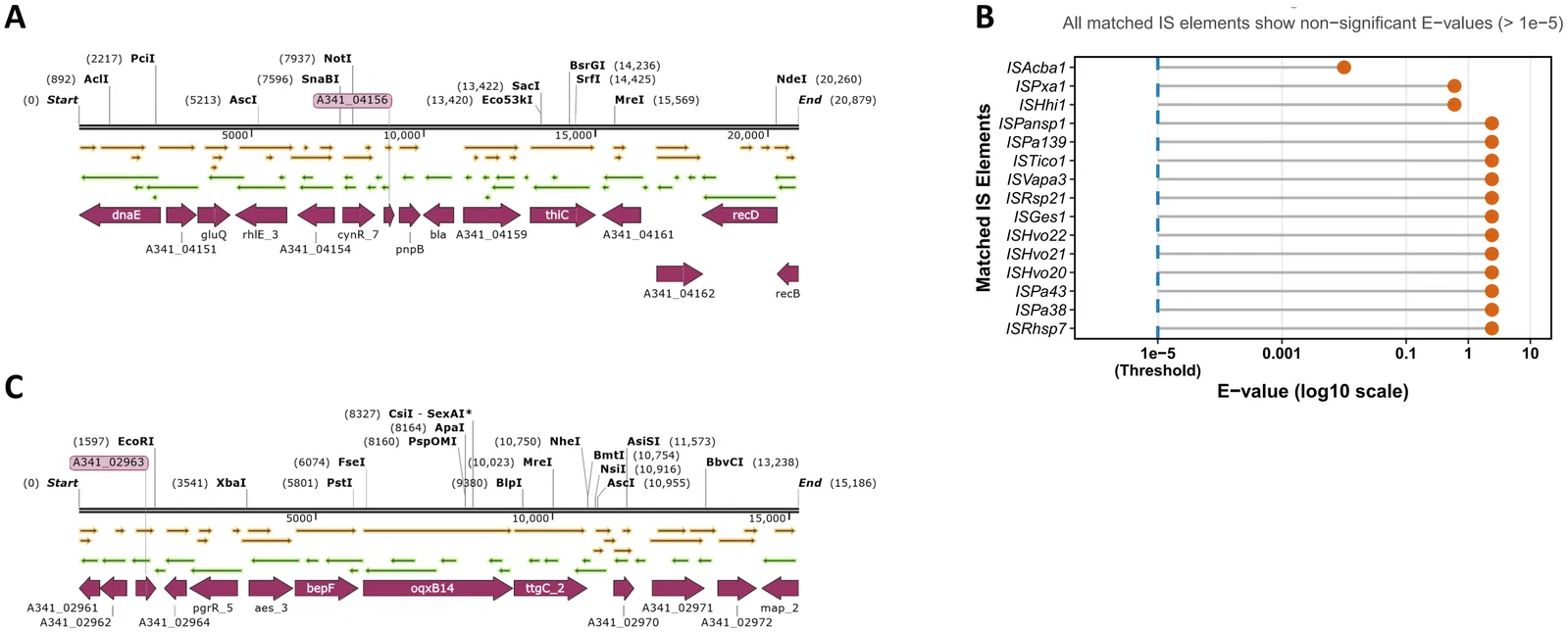
Genetic environment and mobilization analysis of key resistance determinants. **(A)** Linear genomic map of the 20-kb region surrounding the *bla*_OXA-1152_ gene. **(B)** Evaluation of insertion sequences within the *bla*_OXA-1152_ genetic context using ISfinder. **(C)** Genetic organization of the 12-kb region encompassing the intact RND-family efflux pump operon.

Analysis of the efflux-associated locus annotated with *adeF* revealed a complete and conserved RND (Resistance-Nodulation-Division)-type efflux pump organization, consisting of *bepF*-*oqxB14*(*adeF*)-*ttgC_2*, with an upstream LysR-family transcriptional regulator annotated as *pgrR_5* (**Figure 7C**). The overall arrangement was intact in both strains, with no evidence of truncation, fragmentation, or insertional disruption. The PgrR_5 proteins from *P. sputorum*-341 and *P. sputorum*-408 were identical to each other but differed from the database reference sequence (WP_150677733.1) at multiple amino acid positions (**Figure 8**). Notably, two amino acid substitutions (M28L and A42S) were located within the N-terminal HTH DNA-binding domain, which is involved in promoter recognition and transcriptional regulation in LysR-family regulators. The functional significance of these substitutions remains unknown, and their possible effect on downstream efflux pump expression requires transcriptional and functional validation. Moreover, because these substitutions were defined relative to a database reference sequence rather than to a carbapenem-susceptible *P. sputorum* comparator, they may represent lineage-associated polymorphisms rather than resistance-associated regulatory changes.

**Figure 8:**
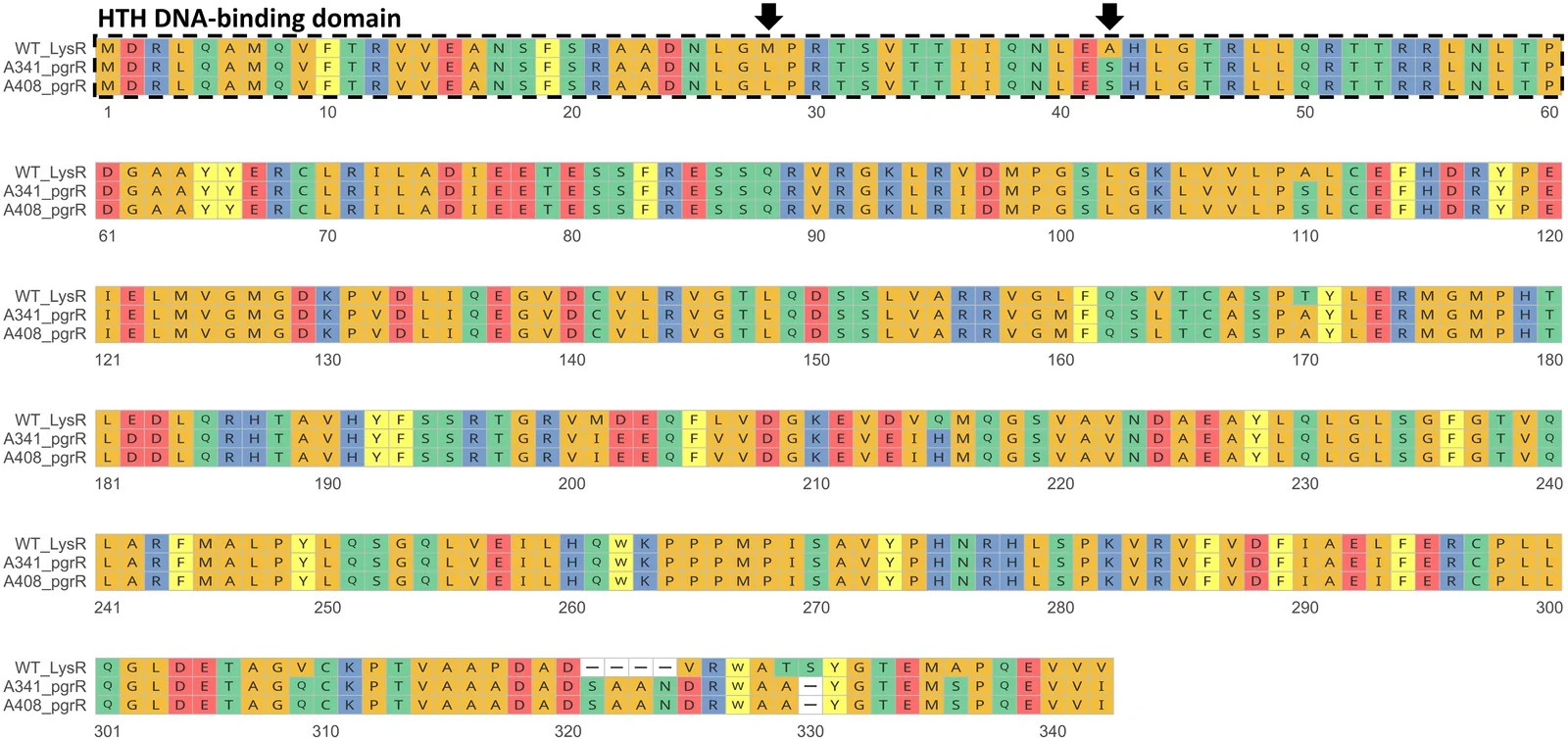
Multiple sequence alignment of the LysR-family transcriptional regulator (PgrR) from *Pandoraea* strains, visualized via ESPript 3.0. Black arrows highlight the critical missense mutations (M28L and A42S) within the HTH domain in the resistant strains.

In addition to beta-lactamase and efflux-associated candidates, both genomes encoded multiple outer-membrane and porin-related proteins as well as PBPs or cell-wall-associated targets. These included porin-family proteins, outer-membrane assembly factors, Omp/OprM-related proteins, and predicted PBPs such as MrdA, DacA, MrcA, and PenA (**Supplementary Table 3**). These features provide additional genomic candidates that could theoretically influence carbapenem entry or target interaction. However, because porin expression, outer-membrane protein abundance, PBP sequence-function relationships, and carbapenem-binding affinities were not experimentally assessed, their contribution to the phenotype remains unresolved and requires further experimental validation. Overall, these findings support a potentially multifactorial interpretation of the carbapenem susceptibility phenotype of the two sequenced isolates, although functional validation is required to establish causality.

## Discussion

This study links a pediatric bloodstream isolate collection with exploratory genomic analysis through a shared microbiological observation: the recurrent meropenem-resistant/imipenem-susceptible phenotype. The clinical analysis established the pediatric bloodstream context and showed that *P. sputorum*-positive blood cultures were associated with mild and nonspecific presentations, whereas WGS of two selected MDR isolates was used to explore candidate genome-encoded determinants underlying this clinically relevant susceptibility pattern. Thus, the genomic analysis was not intended as an independent survey of two isolates, but as a targeted exploratory analysis motivated by the recurrent phenotype identified in the clinical collection.

In recent years, several *Pandoraea* species have increasingly been recognized as opportunistic human pathogens, particularly in patients with CF, and have also been implicated in non-CF infections such as pneumonia and bloodstream infection (Gao et al., 2022; Reshad et al., 2024; Saiman et al., 2024; Chen et al., 2025; Li et al., 2025). Compared with the relatively better-described role of *Pandoraea* in CF-associated respiratory disease, the clinical significance and resistance mechanisms of *P. sputorum* bloodstream infection remain insufficiently characterized, especially in pediatric patients. In this context, our study provides a clinically and genomically integrated analysis of pediatric bloodstream isolates of *P. sputorum*, with particular emphasis on the discordant carbapenem susceptibility phenotype and its potential genomic basis.

Previous reports of *Pandoraea* bloodstream infections have generally emphasized their rarity but potential clinical severity (Wu et al., 2026). Severe invasive infections, including sepsis, multiple organ failure, and fatal outcomes, have been described, particularly in vulnerable or immunocompromised hosts (Stryjewski et al., 2003; Johnson et al., 2004; Pimentel and MacLeod, 2008; Xiao et al., 2019; Bodendoerfer et al., 2021; Itoh et al., 2022; Chen et al., 2025). More recently, Wu et al. performed WGS of 18 P*. sputorum* isolates recovered from patients with bloodstream infections in China and reported a case fatality rate of 27.8%, supporting the view that *P. sputorum* can be associated with high-mortality invasive disease (Wu et al., 2026). In our pediatric cohort, the clinical manifestations were relatively nonspecific. Most patients presented with fever and elevated inflammatory markers, whereas total white blood cell counts were often only modestly elevated. This difference may reflect differences in host population, infection source, disease severity, or the possibility that some positive cultures represented low-grade bacteremia or contamination.

Because *Pandoraea* spp. are environmental non-glucose-fermenting Gram-negative bacilli and may persist in aqueous environments, the possibility of healthcare-associated acquisition, or common-source contamination should be considered when multiple isolates are recovered in a single institution. To address this issue, we reviewed the available clinical hospitalization data. The cases occurred over approximately two years and were distributed across multiple hospital units rather than being restricted to a single ward. Importantly, 32 of 34 blood cultures (94.1%) were collected on the day of admission or within 48 h after admission. This timing pattern argues against typical hospital-onset bloodstream infection for the majority of cases. Although Pediatric Gastroenterology Ward II contributed the largest number of cases, the corresponding isolates were recovered intermittently over a prolonged period, rather than within a short, epidemiologically linked cluster. Taken together, the available epidemiological data do not support a typical short-term single outbreak or point-source healthcare-associated transmission event. However, these observations do not definitively exclude healthcare-associated acquisition, which requires future studies incorporating environmental surveillance and molecular typing methods, such as Fourier-transform infrared spectroscopy (FT-IR spectroscopy), pulsed-field gel electrophoresis (PFGE), core-genome multilocus sequence typing (cgMLST), or WGS of all isolates, to distinguish sporadic bloodstream infection from potential healthcare-associated transmission. In the present study, WGS was performed for only two selected *P. sputorum* isolates. Therefore, the genomic findings should be interpreted as strain-specific observations and should not be extrapolated to all 34 isolates.

This study links a pediatric clinical bloodstream isolate collection with genomic analysis of two selected MDR *P. sputorum* strains. The central observation connecting the clinical and genomic components is the highly conserved carbapenem-discordant phenotype across all 34 bloodstream isolates, characterized by meropenem resistance with retained imipenem susceptibility, which has also been reported or suggested in related *Pandoraea* genomic studies (Schneider and Bauernfeind, 2015; Chen et al., 2025; Zhang et al., 2025). Because the clinical manifestations were nonspecific, the major clinical relevance of this organism lies in accurate microbiological identification, careful antimicrobial susceptibility interpretation, and understanding the genome-encoded mechanisms that may explain this unusual phenotype. The most distinctive microbiological finding of this study was the conserved beta-lactam susceptibility discordance shared by the 34 isolates. The consistent observation of meropenem resistance with imipenem susceptibility, together with cefepime/ceftazidime resistance and relative ceftriaxone susceptibility, across isolates collected throughout the study period suggests that this phenotype may reflect stable, chromosome-encoded mechanisms rather than sporadic acquisition events. This observation is consistent with the carbapenem discordance recently described by Zhang et al., who identified the oxacillinase variant *bla*_OXA-1152_ in 12 P*. sputorum* strains from Wuhan, China (Zhang et al., 2025). In that study, deletion of *bla*_OXA-1152_ reduced meropenem and imipenem MICs by 64-fold and 4-fold, respectively, and reintroduction of the gene restored resistance. Biochemical analyses further showed that OXA-1152 had stronger binding and hydrolytic activity toward meropenem than imipenem. These functional data provide a plausible mechanistic explanation for the meropenem-resistant/imipenem-susceptible phenotype observed in our two sequenced isolates. Our findings extend this observation to pediatric bloodstream isolates and suggest that this unusual carbapenem discordance may be more broadly distributed among clinically invasive *P. sputorum* strains.

OXA-family beta-lactamases appear to be recurrent genome-encoded features in *Pandoraea* species. In a whole-genome analysis of *Pandoraea* strains recovered from CF patients, Bayjanov et al. found that the isolates did not harbor acquired antibiotic resistance genes but encoded an OXA-type beta-lactamase, suggesting that beta-lactam resistance in *Pandoraea* may often be associated with intrinsic or chromosome-encoded determinants rather than classical acquired resistance genes (Bayjanov et al., 2019). Similarly, Wu et al. reported that bloodstream infection-associated *P. sputorum* isolates harbored core genome-encoded OXA-155 beta-lactamase and ceoB efflux pump genes, and displayed multidrug resistance while remaining susceptible to imipenem (Wu et al., 2026). Together with the functional evidence for OXA-1152 reported by Zhang et al. (2025), these studies suggest that different OXA-family beta-lactamases may contribute to species- or lineage-specific beta-lactam resistance phenotypes in *P. sputorum*. In our study, chromosomal *bla*_OXA-1152_ was present in both sequenced isolates, *P. sputorum*-341 and *P. sputorum*-408, and our phylogenetic analysis confirmed that both strains are closely related to the previously characterized *bla*_OXA-1152_-positive strains reported by Zhang et al., supporting the possibility that a similar resistance mechanism operates in the two sequenced isolates. However, it should be emphasized that the direct functional validation in our specific isolates was not performed.

Nevertheless, the resistance phenotype of *P. sputorum* is likely multifactorial. In addition to *bla*_OXA-1152_, our annotation-based screening identified multiple candidate mechanisms potentially related to beta-lactam susceptibility, including efflux-associated systems, outer-membrane or porin-related proteins, and penicillin-binding protein or cell-wall-associated targets. We also identified a complete *adeF*-associated RND efflux locus with amino acid substitutions (M28L and A42S) in the HTH DNA-binding domain of its upstream LysR-family regulator. RND-type efflux systems are well known to contribute to broad-spectrum multidrug resistance in Gram-negative bacteria (Paulsen et al., 1996; Ong et al., 2007; Nikaido and Pagès, 2012; Nikaido and Pagès, 2012; Lorusso et al., 2022), and similar efflux-related determinants have been reported in *P. sputorum* bloodstream infection-associated lineages. Therefore, the *adeF*-associated locus may contribute to the overall MDR background of the two sequenced isolates. However, because RND efflux systems usually affect multiple antimicrobial classes rather than producing highly specific carbapenem discordance, *bla*_OXA-1152_ remains the most plausible candidate for the meropenem-resistant/imipenem-susceptible phenotype. This interpretation should be considered hypothesis-generating, because we did not perform gene knockout, complementation, enzyme kinetic assays, efflux inhibition assays, porin expression analysis, transcriptomic profiling, or PBP functional assays in our isolates.

The phylogenetic context further supports the importance of genome-based analysis for *P. sputorum*. Previous WGS studies of CF-associated *Pandoraea* strains demonstrated that several isolates historically assigned to recognized species, including *P. sputorum*, *P. pnomenusa*, *P. oxalativorans*, and *P. pulmonicola*, may represent novel taxa, highlighting the taxonomic complexity of this genus and the value of genome-based species identification (Bayjanov et al., 2019; Mia et al., 2026). Other comparative genomic analyses have identified environmental or plant-associated isolates as *P. sputorum* using ANI, AAI, MLSA, *rpoB* similarity, and genome-to-genome distance approaches, and have reported selected functional traits such as quorum sensing systems and OXA-type beta-lactamases (Gao et al., 2022). In our analysis, *P. sputorum*-341 and *P. sputorum*-408 clustered with publicly available *P. sputorum* genomes within a relatively conserved phylogenetic background in which resistance-associated and virulence-associated genes were broadly shared. These results suggest that the resistance phenotype observed in the two sequenced isolates may reflect lineage-linked genomic features. However, because WGS was limited to two selected isolates, the population-level distribution of *bla*_OXA-1152_, *adeF*-associated efflux loci, and other candidate resistance determinants among all 34 pediatric bloodstream isolates remains to be determined.

From a clinical standpoint, our findings have implications for both clinical microbiology laboratories and clinicians managing rare Gram-negative bloodstream infections. The discordant carbapenem susceptibility phenotype may be overlooked if *Pandoraea* is misidentified as a more common non-fermenting Gram-negative bacillus or dismissed as a contaminant. We should be aware that meropenem resistance does not necessarily preclude imipenem susceptibility in *P. sputorum*. Therefore, careful species-level identification and complete antimicrobial susceptibility testing are essential for appropriate interpretation. The consistent susceptibility to trimethoprim-sulfamethoxazole observed across all 34 isolates suggests that this agent may represent a potentially effective option for pediatric *P. sputorum* bloodstream infection when supported by susceptibility testing (Schneider and Bauernfeind, 2015; Clinical and Laboratory Standards Institute, 2026). Given the safety limitations of fluoroquinolones in pediatric populations and the preserved susceptibility to imipenem, an empirical or early targeted regimen including imipenem rather than meropenem, with or without trimethoprim-sulfamethoxazole depending on clinical severity and susceptibility results, may be considered. In light of the avibactam-carbapenem synergy reported by Zhang et al. (2025), beta-lactamase inhibitor combinations may also warrant further evaluation for MDR *Pandoraea* infections, although clinical evidence remains limited.

### Limitations

Several limitations should be considered when interpreting the genomic findings. First, WGS was performed on only two selected isolates; therefore, WGS-based conclusions were restricted to these two sequenced isolates and should not be extrapolated to the full collection of 34 isolates. Second, molecular typing was not performed for all isolates, preventing definitive assessment of clonal relatedness or shared genetic background. Third, although candidate resistance-associated genes were identified in the two sequenced isolates, WGS alone cannot confirm their expression, enzymatic activity, or functional contribution to the observed carbapenem susceptibility pattern. Functional validation, such as gene expression analysis, β-lactamase activity testing, gene knockout experiments, and sequencing or typing of additional isolates, is needed to clarify the genetic basis of carbapenem susceptibility discordance in *P. sputorum*.

## Conclusions

This study provides integrated clinical and genomic insights into *P. sputorum* as an underrecognized cause of pediatric bloodstream infection. The 34 isolates recovered from individual patients over approximately 2 years displayed a conserved MDR profile with a distinctive discordant carbapenem susceptibility phenotype. Based on the WGS, comparative genomics, and phylogenetic analysis of two selected isolates, chromosomal *bla*_OXA-1152_ and an *adeF*-associated efflux system with potential regulatory mutations may serve as candidate contributors to this phenotype. From a clinical perspective, trimethoprim-sulfamethoxazole and imipenem (rather than meropenem) may represent viable treatment options for *P. sputorum* bloodstream infection in children. These findings provide a framework for future studies aimed at functional validation of resistance mechanisms, and prospective surveillance of this uncommon pathogen.

## Data Availability

The datasets presented in this study can be found in online repositories. The names of the repository/repositories and accession number(s) can be found below: https://www.ncbi.nlm.nih.gov/genbank/, CP184737.1 https://www.ncbi.nlm.nih.gov/genbank/, CP184738.1.
